# Efficient oligonucleotide probe selection for pan-genomic tiling arrays

**DOI:** 10.1186/1471-2105-10-293

**Published:** 2009-09-16

**Authors:** Adam M Phillippy, Xiangyu Deng, Wei Zhang, Steven L Salzberg

**Affiliations:** 1 Center for Bioinformatics and Computational Biology, University of Maryland, College Park, MD 20742, USA; 2 National Center for Food Safety and Technology, Illinois Institute of Technology, Summit, IL 60501, USA

## Abstract

**Background:**

Array comparative genomic hybridization is a fast and cost-effective method for detecting, genotyping, and comparing the genomic sequence of unknown bacterial isolates. This method, as with all microarray applications, requires adequate coverage of probes targeting the regions of interest. An unbiased tiling of probes across the entire length of the genome is the most flexible design approach. However, such a whole-genome tiling requires that the genome sequence is known in advance. For the accurate analysis of uncharacterized bacteria, an array must query a fully representative set of sequences from the species' pan-genome. Prior microarrays have included only a single strain per array or the conserved sequences of gene families. These arrays omit potentially important genes and sequence variants from the pan-genome.

**Results:**

This paper presents a new probe selection algorithm (PanArray) that can tile multiple whole genomes using a minimal number of probes. Unlike arrays built on clustered gene families, PanArray uses an unbiased, probe-centric approach that does not rely on annotations, gene clustering, or multi-alignments. Instead, probes are evenly tiled across all sequences of the pan-genome at a consistent level of coverage. To minimize the required number of probes, probes conserved across multiple strains in the pan-genome are selected first, and additional probes are used only where necessary to span polymorphic regions of the genome. The viability of the algorithm is demonstrated by array designs for seven different bacterial pan-genomes and, in particular, the design of a 385,000 probe array that fully tiles the genomes of 20 different *Listeria monocytogenes *strains with overlapping probes at greater than twofold coverage.

**Conclusion:**

PanArray is an oligonucleotide probe selection algorithm for tiling multiple genome sequences using a minimal number of probes. It is capable of fully tiling all genomes of a species on a single microarray chip. These unique pan-genome tiling arrays provide maximum flexibility for the analysis of both known and uncharacterized strains.

## Background

Microarrays are well known for their success in studying gene expression [1]. As one of their many other roles, DNA microarrays can also be used to characterize both large-scale and small-scale genetic variations. For instance, array comparative genomic hybridization (aCGH) is commonly used in human cancer studies to genotype cell lines by detecting gene loss and copy number variations [2]. At a finer resolution, microarrays are also used to detect single nucleotide polymorphisms at targeted loci [3]. In addition to human screens, microarrays have been widely used for the detection and genotyping of microbial species. Notably, a viral genotyping microarray [4] was one of the methods used to etiologically link severe acute respiratory syndrome (SARS) to a novel coronavirus [5]. Arrays for the detection and comparative analysis of bacterial genomes have also been developed, including arrays for *Listeria monocytogenes *[6-10], and many other bacterial species. However, these earlier, low-density arrays did not contain enough probes to target the entire genome of the bacterium, and were forced to probe only a small subset of the known genes.

**Figure 1:**
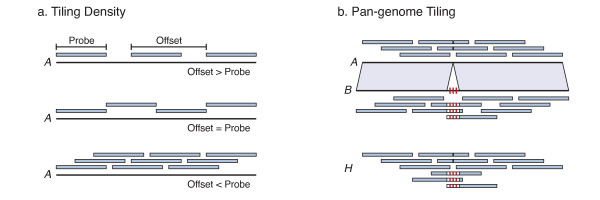
**Illustration of different tiling densities, and an example pan-genome tiling**. Genomes are represented as horizontal lines and probes as colored rectangles. The *offset *between probes is the distance between the start of one probe and the start of the next. (1a) Three different tiling densities are shown for genome *A*. The top figure illustrates a gapped tiling, the middle an end-to-end tiling, and the bottom an overlapping tiling. (1b) A pan-genome tiling is shown for two genomes. Genomes *A *and *B *are identical except for a small insertion in *B*, represented by vertical red bars. Solid blue probes are conserved in both genomes, and probes spanning the insertion event are colored by variant. Set *H *shows the non-redundant set of probes needed to tile the pan-genome including *A *and *B*.

As the density of DNA microarrays increased in recent years, it has become possible to probe the entire genome of an organism in addition to only specific genes. An array providing unbiased coverage of probes across a genome is commonly referred to as a *whole-genome tiling array*. Such arrays have been very successful for genome-scale analysis, including the discovery of novel transcripts, splicing variants, protein binding sites, and polymorphisms [11]. Depending on the offset between adjacent probe locations, whole-genome tilings can be either gapped, end-to-end, or overlapping (Figure 1a).

In the human genome, tiling arrays are designed to probe the genome at evenly spaced intervals. To maximize the expected specificity of the array, repetitive probes must be avoided and experimental conditions, such as melting temperature, equalized. This creates an optimization problem in choosing which sequences should be included on the array [12,13]. In smaller microbial genomes, it is possible to target every position of the genome with overlapping probes, simplifying the design process. For example, extreme high-density arrays can now accommodate 2.1 million variable length probes on a single chip (Roche NimbleGen, Inc). For an average 2 Mb sized bacterial genome and 50 nt probe length, probes can be offset by only a single base-pair and still span the entire genome, generating a coverage of 50×. By tiling the entire genome, some suboptimal probes will be included on the array, but can be identified and corrected for in the analysis. These overlapping arrays are capable of identifying polymorphism at a much finer resolution than gapped arrays.

Tiling arrays have traditionally been constructed based on the genome of a single reference strain and used to locate genomic differences contained in the experimental strains. However, single-genome arrays can only detect and analyze sequences similar to those included on the array, and cannot discover or analyze sequences absent in the reference strain. After the introduction of the pan-genome concept [14,15], it has become increasingly clear that some microbial species contain significant genetic diversity, and it is not suitable to compare against only a single reference strain. The pan-genome hypothesis states that any given species has two sets of genes. First, a set of *core *genes present in all strains that define the species; and second, a set of *dispensable *genes present in only one or a few of the strains that presumably mediate adaptation. A single genome describes the genomic material for a particular strain, but the pan-genome describes the genomic makeup for an entire species. Single reference tiling arrays cannot survey this full diversity. Ideally, an array for analyzing new strains should cover the genomic diversity of the entire pan-genome.

With the explosion in microarray densities, it is now possible to design pan-genome tiling arrays that contain all genomic sequence from the known pan-genome. The simplest strategy is to fully tile the genomes of each strain independently. However, due to similarities between the strains, some sequences would be tiled with excessive redundancy, and this approach would be cost ineffective. Instead, a pan-genome array should aim to minimize costs by using the minimal probe set necessary to target every element of the pan-genome with adequate coverage. The typical approach for targeting multiple strains is to group individual genes into gene families and then probe only the conserved sequences of those families [16-18]. For example, Willenbrock et al. designed an innovative 32 strain *Escherichia coli *pan-genome array by clustering homologous genes based on pairwise alignment similarity [16]. Homology was defined as gene alignments with an E-value < 10^-5^, a bitscore > 55, and alignment coverage of at least 50% of the gene length. For each resulting gene group, a consensus sequence was generated via multiple alignment, and probes were designed to target the most conserved regions of the consensus. The resulting array comprised 224,805 probes, targeting 9,252 gene groups, with a median coverage of 27 probes per gene group.

Targeting only the conserved sequence of gene families is an effective and efficient method for detecting--at a low resolution--the presence and absence of gene families; however, for studies that require a finer resolution, this method omits many potentially significant sequences from the array. Firstly, a slight variation in a gene (e.g. a partial deletion) can be responsible for a significantly different phenotype. By only targeting the conserved portion of gene families, the variable regions responsible for these differences will not be included on the array. Secondly, a gene-centric design includes only coding sequences. Therefore, these designs cannot be used to detect differences in intergenic regions which may include regulatory elements, or used for studies that require a whole-genome tiling, such as transcriptome mapping or chromatin-immunoprecipitation-chip (ChIP-chip) studies. Finally, gene-centric design models depend on an accurate annotation of the genome. If genes have been mis-annotated or omitted from the annotation, such genes cannot be properly represented on the array. This is particularly troublesome for many draft-quality genomes that have highly fragmented sequence assemblies and lack accurate annotations. For these reasons, a whole-genome tiling is preferable for applications that require more flexibility or an unbiased tiling of the genome. However, no methods have been described for efficiently tiling multiple whole-genome sequences.

This paper describes a method for pan-genome tiling array design that both minimizes the number of probes required and guarantees that all sequences in the pan-genome are fully tiled by the array. The prior gene-centric approaches are abandoned in favor of a more concrete, probe-centric approach that relies only on the genomic sequences and not the annotation. To summarize the new approach, let the pan-genome *G *be the set of all genomes from a species, and let *P *be the non-redundant set of all length *k *substrings from *G*. Due to sequence conservation between genomes, a single probe may match to multiple locations (genomes) of the pan-genome. Call these matches the probe *targets*. The *Pan-Tiling *problem is to find a minimum cardinality subset *H *⊆ *P *such that all sequences of *G *are targeted by probes in *H *and no target is offset more than *maxoff *from an adjacent target (or sequence end).

Constructing a full tiling of the pan-genome seems like it would require a large number of probes, but by leveraging the similarities between strains, a reasonably sized probe set can be constructed that fully covers a large pan-genome with adequate redundancy. The key to the strategy is choosing probes that will hybridize to as many of the strains as possible, while using only a necessary amount of probes to cover polymorphisms (insertions, deletions, variants). For example, Figure 1b shows a pan-genome tiling for two miniature genomes, with a *maxoff *of one-third the probe length. Genomes *A *and *B *are identical except for a small insertion in the middle of *B*. Fully tiling both genomes requires a total of 19 probe targets (9 for *A *and 10 for *B*), but probe set *H *illustrates that these 19 targets can be tiled with just 12 probes. Conserved probes are used to tile the left and right of both genomes, and distinct probes are used to tile the two polymorphism variants. This is obviously a simplified example. The problem becomes more difficult as the number of genomes and complexity of polymorphisms increases.

The methods presented in this paper were developed to aid the design of a pan-genome CGH tiling array for *Listeria monocytogenes*--the causative agent of listeriosis and a NIAID category B biodefense agent that is of significant food safety and public health concern [19]. The species of *L. monocytogenes *is composed of three primary genetic lineages (named I, II, and III) that display different capabilities of environmental survival and pathogenic potential to cause human infectious disease [20]. In order to both characterize new strains based on genetic content, and detect polymorphism at a higher resolution in small RNAs (sRNAs) and intergenic sequences, the array was required to cover all pan-genomic sequences with a high density of probes. This bacterial species is particularly well suited for pan-genome array design because there are a remarkable number of strains that have been sequenced. At the time of writing, a total of 20 *L. monocytogenes *complete or draft genome sequences were available, totaling 57.9 Mbp (Table 1). Genomic sequences and annotations were obtained from The National Microbial Pathogen Database Resource (NMPDR) [21]. The sequence conservation for the sequenced strains was computed with Nucmer [22], and ranges between 94% and 99% in nucleotide identity versus the completed *EGD-e *reference strain. Even with such substantial diversity within the species, the PanArray algorithm is able to design a pan-genome tiling covering each genome at more than twofold coverage using only 385,000 50-mer probes. A similar density tiling for a single *L. monocytogenes *strain would require 125,000 probes, meaning the PanArray design covers 20× more genomes using only 3× more probes. A description of this design, along with array designs for six other bacterial pan-genomes is given in the Results section.

**Table 1:** Genomic sequences included on the *Listeria monocytogenes *pan-genome tiling array.

Strain	Lineage	Serotype	Bases	Contigs	Genes*	EGD-e %Idy
EGD-e	II	1/2a	2,944,528	1	3,002	100
LO28	II	1/2c	2,910,810	529	5,078	99.3
FSL F2-515	II	1/2a	2,586,267	1,415	NA	98.41
FSL J2-003	II	1/2a	2,878,206	406	4,686	98.22
1/2a F6854	II	1/2a	2,950,285	133	3,028	98.01
FSL N3-165	II	1/2a	2,886,689	33	2,963	97.52
J2818	II	1/2a	2,971,223	38	3,270	97.19
F6900	II	1/2a	2,958,319	35	3,333	97.15
J0161	II	1/2a	3,051,828	51	3,252	97.09
10403S	II	1/2a	2,866,709	32	2,944	96.9
FSL J2-064	I	1/2b	2,899,431	327	3,914	94.69
4b H7858	I	4b	2,972,254	181	3,187	94.54
FSL J1-175	I	1/2b	2,902,346	357	4,559	94.49
FSL N1-017	I	4b	2,857,865	77	3,465	94.3
HPB2262	I	4b	3,006,068	75	3,319	94.01
FSL J1-194	I	1/2b	2,986,227	44	3,792	93.98
4b F2365	I	4b	2,905,187	1	2,987	93.87
FSL R2-503	I	1/2b	3,001,696	54	4,863	93.73
FSL J2-071	IIIA	4c	3,149,923	46	3,789	93.28
FSL J1-208	IIIB	4a	2,260,760	1,494	NA	92.84

Sequences and annotations were obtained from NMPDR. The final column shows the nucleotide identity of a whole-genome alignment versus strain EGD-e. *Number of annotated protein coding genes and RNAs reported by NMPDR at the time of this study.

## Methods

The general strategy of the PanArray design algorithm is best summarized by analogy to the well-known *Minimum Hitting Set *problem in computer science [23,24]. Let *P *be a set of *n *points and *F *= {*P*_1_, *P*_2_,..., *P*_*m*_} be a family of *m *subsets of *P*. *Minimum Hitting Set *is the problem of selecting the minimum cardinality subset *H *⊆ *P *such that *H *contains at least one element from each subset in *F*. Although finding a minimum hitting set is known to be NP hard, it is a well studied problem and efficient approximation algorithms are known.

To see the similarities between the *Pan-Tiling *and *Minimum Hitting Set *problems, let the sequence *G *be a concatenation of all the genomes from a species, and let *W *= {*w*_1_, *w*_2_,..., *w*_*m*_} be the set of *m *intervals that results from segmenting *G *into non-overlapping, end-to-end, length *l *windows. Let *P *be the non-redundant set of length *k *substrings from *G*. A probe candidate *p *∈ *P *is said to *hit *a window *w *∈ *W *if a match between *p *and a substring of *G *begins in the interval *w*. Let *P*_*i *_⊆ *P *be the subset of probes that hit the window *w*_*i*_, and *F *= {*P*_1_, *P*_2_,..., *P*_*m*_} for the *m *windows of *W*. A minimum hitting set *H *of *F *is a minimum cardinality subset of probes *H *⊆ *P *such that every window of the pan-genome is hit by at least one probe in *H*. Therefore, finding *H *effectively tiles the entire pan-genome using a small number of probes.

### Window and probe indexing

Windowing the genome simplifies the *Pan-Tiling *problem by casting it is a *Minimum Hitting Set *problem, and at the same time enforces the *maxoff *constraint. Because each window is forced to contain at least one target, any two adjacent targets cannot be separated by more than twice the window length. Therefore, the window length is equal to one half *maxoff*. For example, given a maximum offset of 2*l*, windows are marked off every *l *bases of the pan-genome--with the first window *w*_1 _covering the interval [1, *l*], and the second window *w*_2 _covering [*l*+1, 2*l*], and so on. Assuming one target is chosen per window, and the target locations are evenly distributed within windows, the average distance between adjacent targets is expected to be equal to the window length. For a window length *l*, equal to the probe length *k*, the resulting depth of coverage averages one, because the probes are spaced *k *bases apart on average. For any other window length *l*, the resulting depth of coverage *c *is expected to be *c *≈ *k*/*l*. The extreme case being *l *= 1, which results in exactly *k*-fold coverage because a probe must hit every position in *G*.

To solve the *Minimum Hitting Set *problem, once the pan-genome is discretized into a set of windows, each window must be mapped to the set of probe hits it contains. As before, a probe *p *hits a window if a match between *p *and *G *begins within the window's interval. Thus far exact matches have been assumed, but a match can be defined by any criteria necessary for efficient hybridization. To help reduce probe redundancy, the PanArray implementation can optionally use inexact matches containing a single mismatch. Any suitable *k*-mer indexing algorithm can be utilized for this phase, but allowing for mismatches can be computationally expensive. The implementation uses a fast, but memory intensive, compressed keyword tree for indexing all probe hits. Alternatively, a slower, but memory efficient, hashing scheme would also work. To index the 1-mismatch hits, each probe's 3*k *possible 1-mimsatch permutations are added to the index as well. The result of the indexing is a list of positions and windows for all *k*-mers of the pan-genome (the probe candidates). At this stage, the final list of probe candidates may be manually filtered based on typical criterion such as melting temperature, GC content, secondary structure, etc. For ungapped tilings, it is impossible to avoid suboptimal probes. However, highly repetitive probes can be identified by the number of genomic positions they map to, and should be discarded if they threaten to confound the array analysis (e.g. by affecting normalization). Alternatively, the input sequences may be masked prior to *k*-mer indexing to avoid repetitive or unwanted sequence altogether.

For CGH arrays, each probe is considered equivalent to its reverse complement, but for expression or transcriptome arrays, forward and reverse strand probes must be considered independently. Probe matches are listed on the strand on which they appear, so for single-stranded samples, the sequence to be synthesized for the array will need to be reversed complemented. For DNA tiling arrays it is helpful to assume the sample will be double-stranded so that genomic inversions in one or more of the strains do not have to be tiled separately.

### Probe selection

As detailed above, selecting a minimum probe set for tiling *S *is equivalent to finding the minimum hitting set of *P*. As before, *W *is the windowed pan-genome. Let *W*_*p *_be the subset of windows hit by probe *p*, and *U *be the set of currently uncovered windows. Let a window hit by at least one probe be termed as *covered*, and the *coverage *of a probe be the number of windows it hits |*W*_*p*_|. A naive algorithm for finding a small hitting set *H *is to choose, for each uncovered window, a probe hitting the window that also hits the most other windows. The idea being that choosing probes with the highest coverage will minimize the total number of probes necessary to cover all windows. However, this approach does not properly account for the probe coverages. Only a single probe is needed to cover a window, so after selecting a probe *p*, all other probes that hit a window in *W*_*p *_will see their effective coverage reduced. Take for instance two probes *p *and *q *that hit the exact same set of windows. Choosing *p *reduces the effective coverage of *q *to zero, because all of *q*'s windows have already been covered by *p*. Let the *residual coverage r*_*p *_of a probe be the effective coverage after some other set of probes have already been chosen (*r*_*p *_= |*W*_*p *_∩ *U*|).

A greedy algorithm first suggested by Johnson [25] improves on the naive approach by allowing to reconsider the residual coverage of probes after each iteration. This algorithm has since been shown to be essentially a best-possible approximation for the *Minimum Hitting Set *problem [26]. When adapted for the current problem, the algorithm chooses, while uncovered windows remain, the probe that hits the most currently uncovered windows. The Greedy PanArray Algorithm is:

   **Greedy PanArray Algorithm**

      *H *= Ø

      *U *= *W*

      **while ***U *≠ Ø

         **select **

            *U *← *U *- *W*_*p*_

            *H *← *H *∪ {*p*}

      **return ***H*

The algorithm itself is straightforward, but it must be carefully implemented to run efficiently. It is infeasible to recompute the residual coverage |*W*_*p *_	∩ *U*| for all *W*_*p *_during each iteration, because both *P *and *W *can be on the order of millions for a large pan-genome. To avoid this complexity, the PanArray implementation exploits a property of the residual coverages that allows it to recompute only a few values at each iteration. Note that for any *p*, its residual coverage *r*_*p *_can never increase. A probe's coverage either remains the same, or decreases because one of its windows was hit by the prior iteration. Therefore, instead of recomputing all residuals after each iteration, it is sufficient to maintain a priority queue of residual coverages and only update stale values at the front of the queue.

At the start of the algorithm, all initial coverages are inserted into the queue. To maintain the priority queue after a new probe is chosen, all residual coverages are considered invalid. During the next iteration, a new *r*_*p *_value is computed for the front of the queue, marked as valid, and reinserted into the queue. This process is repeated until a valid residual returns to the front of the queue. Often, newly computed residuals will return quickly to the head of the queue before the others have been updated. At this point it is unnecessary to update any other residuals because their new values cannot be greater than their current value. Therefore, the head of the queue must be the updated maximum. This lazy evaluation of the residuals avoids many unnecessary computations and drastically improves the performance of the algorithm. The greedy algorithm without this speedup takes days to complete, but with the speedup runs in a matter of seconds.

### Probe annotation

The flexibility of the PanArray design algorithm is a result of its probe-centric approach. Because it does not require any identification or clustering of genes, the design is independent of any genome annotation. Therefore, instead of building the annotation into the design of the array, the annotation can be mapped onto the array after the design. Most importantly, this strategy allows for intergenic sequence and unannotated genomes to be included on the array, and annotation updates to be incorporated as they become available. For example, after the *L. monocytogenes *array had been designed (see Results), over 40 new sRNAs were discovered in *Listeria *[27]. Neatly, the sequences of each had already been tiled by the array design, and the updated annotation was easily remapped onto the array. As another example, the gene counts provided by NMPDR in Table 1 are inconsistent and vary between 3,000 and 5,000 genes per genome, suggesting considerable annotation error. Uncoupling the array design from the annotations removes any possibility that annotation errors will affect the design.

Included with the final probe set *H *is the list of locations on the pan-genome that each probe matches. If the genome sequence is updated, the location information can be easily recovered by remapping the probes to the genome using a matching tool such as MUMmer [22] or Vmatch [28]. To annotate the array, probes are mapped to all annotation features with a coinciding location. The result is a many-to-many mapping with each feature being targeted by multiple probes, and a single probe possibly targeting multiple features (e.g. conserved genes between strains). With this mapping, all probes targeting a specific gene in the pan-genome can be quickly recovered.

## Results

### *Listeria monocytogenes *pan-genome array

**Figure 2:**
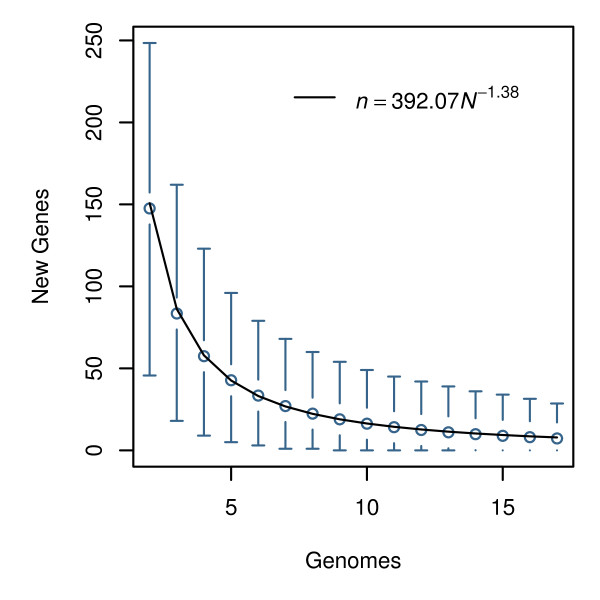
**The number of new genes *n *predicted to be discovered with the addition of an *N*^*th *^*Listeria monocytogenes *genome sequence**. A power law fit to the simulated data is given by the solid curve. The circles represent the mean value for each *N*, and error bars show the 90% confidence intervals.

As suggested in the Introduction, *L. monocytogenes *is a good candidate for constructing a pan-genome tiling array because the species has been widely sequenced, with 20 complete or draft genome sequences available. To confirm that the sequenced genomes contain the majority of *L. monocytogenes *genetic diversity, the pan-genome size was estimated using the methods of Tettelin et al. [15] as implemented in the Ergatis package [29]. Seventeen of the eighteen *L. monocytogenes *genomes listed as annotated by NMPDR in Table 1 were used in the analysis (strain *1/2a F6854 *was unavailable at the time). According to the cited method, the addition of an *N*^th ^genome was simulated by searching the annotated genes of each genome against all possible permutations of *N*-1 other genomes. Genes without a match over 50% protein similarity for at least 50% of their length were recorded as "new". The number of new genes *n *expected to be discovered in the *N*^th ^sequenced genome was modeled by the power law *n *= *κN*^-*α*^, and the parameters κ and α were estimated from the data via non-linear least squares regression using the R function *nls *[30]. The regression was performed on the full set of over 1 million data points. A power law model was found to fit the *L. monocytogenes *data better than the originally proposed exponential model. This agrees with a recent suggestion that a power law is a more appropriate model of the pan-genome phenomenon [31]. The estimated number of undiscovered genes is shown in Figure 2. The power law exponent α was found to be 1.38 ± 0.002, suggesting that the *L. monocytogenes *pan-genome is closed (i.e. has a finite number of genes), and the sequencing of more genomes would eventually sample the entire set of dispensable genes. Therefore, it appears the vast majority of *L. monocytogenes *genes have been sequenced and are included on the array. This model predicts that the addition of a 21^st ^genome to Table 1 would yield only ~ 6 new genes. However, only a single lineage III genome was included in this analysis, so this prediction might be artificially low for a new lineage III strain. The sole lineage III strain analyzed (*FSL J2-071*) contains 31 genes absent in any of the lineage I and II strains.

To capture the full diversity of *L. monocytogenes*, all 20 genomes listed in Table 1 were included in the design, with a combined sequence length of 57,946,621 bp and a total of 65,431 annotated genes. To avoid tiling low quality or contaminant sequence, contigs less than 2 Kbp in length were discarded--reducing the tiled sequence length to 54,810,759 bp. The design was constrained to a 385,000 feature NimbleGen array with a probe length of 50 nt. Because hybridization of a 50-mer probe will tolerate a few mismatches, probes differing by a single mismatch were considered equivalent during the design phase. The window length was set to 24 bp, enforcing a maximum target offset of 48, an expected depth of coverage of about 50/24 = 2.08×, and resulting in approximately 2.3 million windows. These parameters guarantee that every base-pair of the pan-genome will be covered by at least one probe, since the maximum offset is less than the probe length.

**Figure 3:**
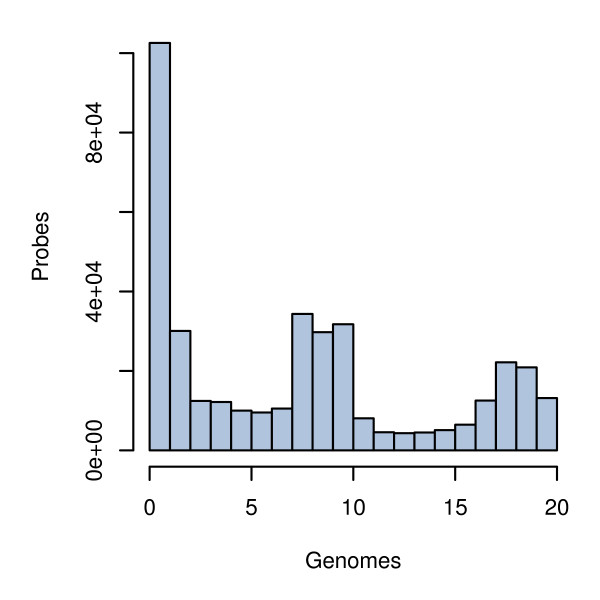
**Histogram of probe reuse for *Listeria monocytogenes***. The number of genomes targeted by each 50-mer probe is given on the horizontal axis. Targets may contain up to one mismatch to the probe.

To cover each window, the PanArray algorithm selected 373,389 distinct probes mapping to 2,893,387 positions in the pan-genome. On average, each probe in the design targets about 8 different positions in the pan-genome. Rather than being repeated sequences within the same genome, these different locations most often refer to a conserved locus in multiple strains (Figure 3). Interestingly, the degree of probe reuse corresponds well with the known evolutionary relationship of the strains. Included on the chip are 8 genomes from lineage I, 10 from lineage II, and 2 from lineage III. This would suggest that the peak at *Genomes *= 1 in Figure 3 is for strain-specific probes; the peaks around 2 and 9 are for lineage-specific probes; and the peak around 20 is for species-specific probes that are conserved in all 20 *L. monocytogenes *genomes.

Because this is a dense tiling of the entire genome, it was unnecessary to optimize probes for uniqueness, as is done in standard expression arrays with only a few probes per gene. Probes were screened for repetitive sequences, but the *L. monocytogenes *strains were found to contain few repeats. The most repetitive 15-mer occurs only 28 times per genome, and the most repetitive 50-mer probe used in the design targets a "cell wall surface anchor protein" family and occurs a maximum of 16 times per genome. Altogether, 99.2% of the probes target at most one location per genome.

**Figure 4:**
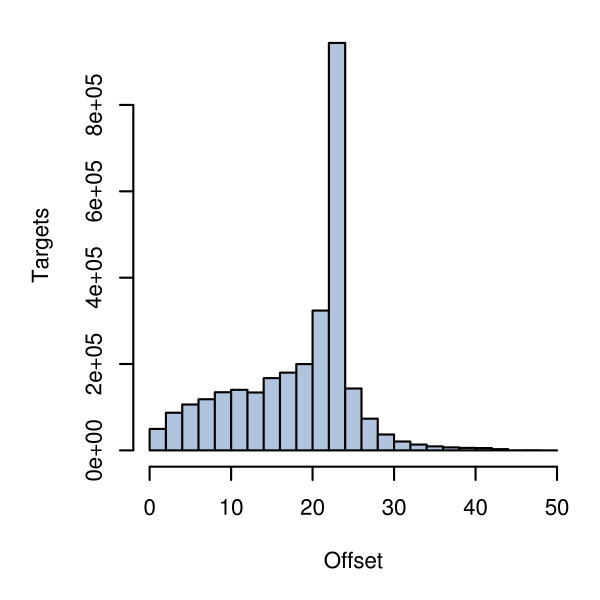
**Histogram of offsets between adjacent probe targets in *Listeria monocytogenes***. The offset between two adjacent probe targets is given on the horizontal axis. Targets may contain up to one mismatch to the probe.

To augment the original PanArray design, an additional 228 negative control probes were added to the array, chosen from *Bacillus spp*., which is a known cohabitant of *Listeria*. The negative control probes were chosen to be specific to *Bacillus spp*. using the Insignia genomic signature design pipeline [32]. The remaining 11,838 features on the array were filled by selecting individual probes to supplement the lowest coverage regions of the design. All probes were checked to conform to NimbleGen design specifications, and a few probes were trimmed to meet synthesis cycle limits. The resulting *L. monocytogenes *pan-genome array has an average depth-of-coverage of 2.65×, with a median probe offset of 21 bp, and a modal offset equal to the window length of 24 bp. The full distribution of probe offsets is given in Figure 4. As expected, the average offset is equal to the window length (24 bp). The uneven distribution and pronounced mode is the caused by non-random tie breaking. In the case of a conserved sequence, where every probe hits the same number of genomes, the first probe of the window is always chosen. Also, the heavy left tail indicates that many windows are covered by more than one probe and the solution that is slightly denser than expected (2.65× actual vs. 2.08× expected). This may be a consequence of the sequence composition, or may indicate a non-optimal solution. Finally, the majority of targeted sequences exactly match their probe (75%) and the remainder match with a single mismatch (25%).

The performance gain of PanArray over more naive methods is significant. For instance, selecting a single probe from each window requires roughly 2.3 million probes. The slightly more principled naive algorithm, that does not recompute residual coverages, chooses 1,739,242 probes, but is still well over the 385,000 probe limit. The Greedy PanArray algorithm meets this limit and vastly outperforms the other methods--requiring only 373,389 probes to cover the entire pan-genome. With the lazy evaluation speedup, the PanArray algorithm is also comparable in runtime to the naive algorithms. On a single 2.4 GHz processor, the naive algorithm took 29 seconds; the greedy algorithm *without *lazy evaluation was terminated without completing after a few days; and the Greedy PanArray algorithm *with *lazy evaluation took only 130 seconds. The runtime for the final design process was dominated by building the *k*-mer index, which required 84 minutes using a compressed keyword tree.

### Design analysis for additional pan-genomes

Using PanArray, additional arrays were designed for a total of seven bacterial pan-genomes, for which a large number of genomes have been sequenced. The additional species include: *Francisella tularensis*, *Staphylococcus aureus*, *Bacillus anthracis*, *Vibrio cholerae*, *Burkholderia pseudomallei*, *Escherichia coli*, and *Shigella spp*. Due to their high similarity, *E. coli *and *Shigella spp*. were considered as a single pan-genome. To facilitate easy comparison, all designs were created with a window length of 25 bp, a probe length of 50 nt, and allowing for probes to contain a single mismatch to their target. As with the *L. monocytogenes *design above, draft genomes were included, but contigs less than 2 Kbp were discarded. The results are given in Table 2. Probe "reuse" is measured in the average number of targets per probe. It is rare for a 50-mer probe to match to more than one location per genome, so the number of targets per probe is roughly equivalent to the average number of genomes that a probe matches.

**Table 2:** Number of probes selected by PanArray to tile various bacterial pan-genomes.

Species	Strains	Avg. Length (Mbp)	Pan Length (Mbp)	Targets	Probes	Reuse
*F. tularensis*	14	1.88	26.29	1,355,504	121,312	11.2 (0.80)
*S. aureus*	14	2.88	40.38	2,006,144	200,999	10.0 (0.71)
*B. anthracis*	9	5.48	49.29	2,230,870	246,947	9.0 (0.99)
*L. monocytogenes*	20	2.74	54.81	2,832,489	358,688	7.9 (0.39)
*V. cholerae*	15	3.87	58.09	3,017,198	346,447	8.7 (0.58)
*B. pseudomallei*	20	6.72	134.31	6,755,234	491,231	13.8 (0.69)
*E. coli/Shigella*	29	4.96	143.72	8,210,679	674,697	12.2 (0.42)

*Avg. length *is the average genome length for a species. *Pan length *is the sum of all genome lengths for a species. *Targets *is the total number of locations targeted by the probes. A single probe may target multiple genomes in the species. *Reuse *is the average number of targets per probe, and a normalized reuse is given in parentheses as the reuse divided by the number of genomes.

The highly conserved species of *B. anthracis *exhibits near perfect probe reuse. Almost every *B. anthracis *probe matches all of the included strains; therefore, the number of probes required to tile the nine sequenced strains is nearly the same as is required to tile one strain. This is because the pan-genome of *B. anthracis *is closed and the strains are highly conserved at the nucleotide level (usually containing only a few SNPs per strain). Adding successive *B. anthracis *strains to the array would increase the required number of probes very gradually.

**Figure 5:**
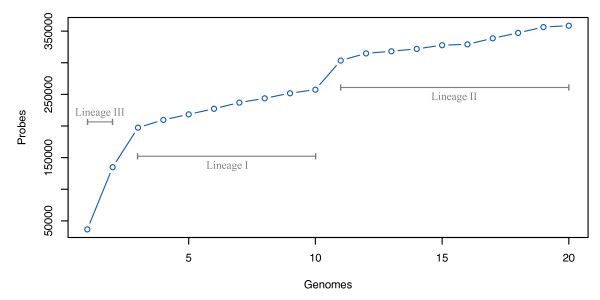
**Number of probes required by PanArray to tile the *Listeria monocytogenes *pan-genome with the successive addition of each genome**. Genomes are added by order of lineage and the design recomputed after each addition.

In contrast, *L. monocytogenes *has the lowest degree of probe reuse, with each probe targeting on average only 39% of the included strains. This is a reflection of the diversity of strains that have been sequenced and the low level of nucleotide conservation between strains, with some strains differing by as much as 8% (see Table 1). Any SNP rate of higher than 2% (1 per 50 bp) exceeds the 1 mismatch threshold per probe and requires additional probes to target the divergent sequence. However, as more variants are added to the array, the addition of each successive genome requires fewer new probes than the last, on average. Figure 5 shows this relationship for the *L. monocytogenes *strains. Successive strains are added by order of lineage, from the bottom of Table 1 to the top, and the design is recomputed at each step. There are pronounced jumps in the number of probes required when the first of a new lineage is added, but the number of probes needed to tile the rest of the lineage quickly levels off.

*Escherichia coli *and *Shigella spp*. form the largest pan-genome currently sequenced, totaling over 144 Mbp of genomic sequence. Even for a pan-genome of this size and diversity, PanArray effectively tiles all sequences at an average of 2× coverage using only 674,697 probes--well below the maximum number of probes available on current arrays. The *B. pseudomallei *pan-genome is roughly equivalent in total number of pan-genome bases, but requires considerably fewer probes because of higher probe reuse. Due to the large number of sequenced genomes and relatively high similarity between strains, the *B. pseudomallei *design exhibits the highest probe reuse factor of all the designs (13.8×). Creating a 2× coverage tiling by choosing one probe every 25 bp would require roughly 5.4 million probes for the *B. pseudomallei *pan-genome, but PanArray was able to create a 2.5× tiling of the same pan-genome with only 491,231 probes.

### Implementation and availability

The PanArray algorithm was implemented in C++, and the source code is freely available at http://www.cbcb.umd.edu/software/panarray. The *Listeria monocytogenes *array design described above is available from the Gene Expression Omnibus [33] under GEO accession number GPL8942.

## Discussion

The PanArray algorithm described above is ideal for high-density tilings of overlapping or closely spaced probes. The Results section has shown that this algorithm is applicable for all currently available bacterial pan-genomes. However, if the maximum number of probes is limited, or the genome size is extremely large, it may be necessary to design a tiling with gaps between the probe targets (i.e. a maximum offset greater than the probe length). In this case, it is necessary to choose unique probes that avoid unwanted cross hybridization between repetitive sequences within the genome. To achieve this, repetitive probes can be filtered, or the coverage scores used in the PanArray algorithm can be weighted to penalize repetitive probes. For example, probe coverage can be redefined as the number of genomes a probe targets, rather than the number of windows, and probes targeting multiple windows in the same genome can be appropriately down-weighted. In many cases, probes within the same window will share the same coverage score, and rules can be applied for breaking the tie and choosing the most reliable probe. Similar schemes could be devised to favor probes with any other desirable criteria.

Array analysis of aCGH experiments is typically conducted on signal ratios between a reference and experimental hybridization. Duplications or deletions in the experimental samples are evident as non-zero values of the log ratio of the two normalized signals. So-called *segmentation *algorithms examine this log ratio across multiple positions in reference sequence to determine the boundaries of the variations [34,35]. The most accurate methods consider not just individual probes, but a context of probes around a genomic location, and can identify even small polymorphisms between the strains. These analyses require both a reference signal and a reference coordinate system on which the probes are tiled. Usually a whole-genome tiling is constructed for a single reference strain, but because PanArray provides a whole-genome tiling for every reference strain included in the array, the same array design can be used to perform segmentation analysis against any reference strain on the array.

In addition to segmentation analysis versus a reference genome, a pan-genome array makes it possible to analyze uncharacterized strains in the context of the entire pan-genome. In some cases, it is preferable to use a multi-strain control [36], but depending on the number of genomes, it can be impractical to co-hybridize all reference strains included on the chip. In these cases, traditional segmentation or log-ratio analysis must be replaced by a method that does not require a reference hybridization signal. For gene-level analysis, direct analysis of the individual probe intensities provides comparable sensitivity and specificity versus segmentation analysis [16], and various methods have been developed that operate independently of a signal ratio [16,37,38]. A probe-based approach provides the most flexibility for pan-genome array analysis, because each probe can be individually scored based on its own intensity, and the genes can be classified based on the aggregated scores of the individual probe scores without the need for a control hybridization.

Pan-genome tiling arrays have all the applications of single-strain tiling arrays, but with enhanced flexibility and the ability to analyze previously uncharacterized strains. Pan-genome aCGH offers an economical alterative to sequencing for determining the genomic makeup of uncharacterized strains in a species and explaining the causative factors of phenotypic differences between strains. Probe based methods, like microarray, are especially well suited for situations where sequencing is inefficient because there is a low abundance of target DNA and a high abundance of background DNA intermixed. For example, applications such as real-time pathogen detection, surveillance, and diagnostics require a known sequence of DNA to be targeted from a vast environment [32,39,40]. A pan-genome array could be used for the detection and genotyping of pathogens from a large environment, without needing to isolate the individual cells. Pan-genome arrays could also be used to capture all species- or locus-specific genomic material from an environment, which could then be directly processed or sequenced separately from the metagenome. Microarray based genomic capture has already been applied to targeted human resequencing as an efficient means of enriching for desired sequencing templates [41-43].

## Conclusion

Without the need for sequencing additional genomes of the same species, pan-genomic aCGH has become an increasingly popular and cost-effective approach to compare and characterize genomic contents of unknown bacterial isolates. Prior multi-strain arrays have targeted the conserved sequences of gene families, or a selected group of polymorphisms; therefore, providing only partial coverage of the pan-genome. PanArray is a probe selection algorithm capable designing a tiling array that fully covers all genomes of a species using a minimal number of probes. The viability of this method is demonstrated by array designs for seven different bacterial pan-genomes, each of which can fit on a single microarray slide. By constructing an unbiased tiling of all known sequences, these unique pan-genome tiling arrays provide maximum flexibility for the analysis, detection, or capture of genomic material for entire species.

## Authors' contributions

AMP conceived the problem, designed and implemented the algorithm, performed the analyses, and wrote the manuscript. XD contributed to the design and analysis, and edited the manuscript. WZ and SLS helped conceive the problem, edited the manuscript, and coordinated the project. All authors read and approved the final manuscript.

## References

[B1] Schena M Shalon D Davis RW Brown PO Quantitative monitoring of gene expression patterns with a complementary DNA microarray Science 1995 270 5235 467 470 10.1126/science.270.5235.467 7569999

[B2] Pinkel D Segraves R Sudar D Clark S Poole I Kowbel D Collins C Kuo WL Chen C Zhai Y High resolution analysis of DNA copy number variation using comparative genomic hybridization to microarrays Nat Genet 1998 20 2 207 211 10.1038/2524 9771718

[B3] Wang DG Fan JB Siao CJ Berno A Young P Sapolsky R Ghandour G Perkins N Winchester E Spencer J Large-scale identification, mapping, and genotyping of single-nucleotide polymorphisms in the human genome Science 1998 280 5366 1077 1082 10.1126/science.280.5366.1077 9582121

[B4] Wang D Coscoy L Zylberberg M Avila PC Boushey HA Ganem D DeRisi JL Microarray-based detection and genotyping of viral pathogens Proc Natl Acad Sci USA 2002 99 24 15687 15692 10.1073/pnas.242579699 12429852 PMC137777

[B5] Ksiazek TG Erdman D Goldsmith CS Zaki SR Peret T Emery S Tong S Urbani C Comer JA Lim W A novel coronavirus associated with severe acute respiratory syndrome N Engl J Med 2003 348 20 1953 1966 10.1056/NEJMoa030781 12690092

[B6] Volokhov D Rasooly A Chumakov K Chizhikov V Identification of Listeria species by microarray-based assay J Clin Microbiol 2002 40 12 4720 4728 10.1128/JCM.40.12.4720-4728.2002 12454178 PMC154633

[B7] Doumith M Cazalet C Simoes N Frangeul L Jacquet C Kunst F Martin P Cossart P Glaser P Buchrieser C New aspects regarding evolution and virulence of Listeria monocytogenes revealed by comparative genomics and DNA arrays Infect Immun 2004 72 2 1072 1083 10.1128/IAI.72.2.1072-1083.2004 14742555 PMC321639

[B8] Call DR Borucki MK Besser TE Mixed-genome microarrays reveal multiple serotype and lineage-specific differences among strains of Listeria monocytogenes J Clin Microbiol 2003 41 2 632 639 10.1128/JCM.41.2.632-639.2003 12574259 PMC149708

[B9] Borucki MK Kim SH Call DR Smole SC Pagotto F Selective discrimination of Listeria monocytogenes epidemic strains by a mixed-genome DNA microarray compared to discrimination by pulsed-field gel electrophoresis, ribotyping, and multilocus sequence typing J Clin Microbiol 2004 42 11 5270 5276 10.1128/JCM.42.11.5270-5276.2004 15528725 PMC525159

[B10] Zhang C Zhang M Ju J Nietfeldt J Wise J Terry PM Olson M Kachman SD Wiedmann M Samadpour M Genome diversification in phylogenetic lineages I and II of Listeria monocytogenes: identification of segments unique to lineage II populations J Bacteriol 2003 185 18 5573 5584 10.1128/JB.185.18.5573-5584.2003 12949110 PMC193770

[B11] Mockler TC Chan S Sundaresan A Chen H Jacobsen SE Ecker JR Applications of DNA tiling arrays for whole-genome analysis Genomics 2005 85 1 1 15 10.1016/j.ygeno.2004.10.005 15607417

[B12] Bertone P Trifonov V Rozowsky JS Schubert F Emanuelsson O Karro J Kao MY Snyder M Gerstein M Design optimization methods for genomic DNA tiling arrays Genome Res 2006 16 2 271 281 10.1101/gr.4452906 16365382 PMC1361723

[B13] Graf S Nielsen FG Kurtz S Huynen MA Birney E Stunnenberg H Flicek P Optimized design and assessment of whole genome tiling arrays Bioinformatics 2007 23 13 i195 204 10.1093/bioinformatics/btm200 17646297 PMC5892713

[B14] Medini D Donati C Tettelin H Masignani V Rappuoli R The microbial pan-genome Curr Opin Genet Dev 2005 15 6 589 594 10.1016/j.gde.2005.09.006 16185861

[B15] Tettelin H Masignani V Cieslewicz MJ Donati C Medini D Ward NL Angiuoli SV Crabtree J Jones AL Durkin AS Genome analysis of multiple pathogenic isolates of Streptococcus agalactiae: implications for the microbial "pan-genome" Proc Natl Acad Sci USA 2005 102 39 13950 13955 10.1073/pnas.0506758102 16172379 PMC1216834

[B16] Willenbrock H Hallin PF Wassenaar TM Ussery DW Characterization of probiotic Escherichia coli isolates with a novel pan-genome microarray Genome Biol 2007 8 12 R267 10.1186/gb-2007-8-12-r267 18088402 PMC2246269

[B17] Feng S Tillier ER A fast and flexible approach to oligonucleotide probe design for genomes and gene families Bioinformatics 2007 23 10 1195 1202 10.1093/bioinformatics/btm114 17392329

[B18] Chung WH Rhee SK Wan XF Bae JW Quan ZX Park YH Design of long oligonucleotide probes for functional gene detection in a microbial community Bioinformatics 2005 21 22 4092 4100 10.1093/bioinformatics/bti673 16159916

[B19] Farber JM Peterkin PI Listeria monocytogenes, a food-borne pathogen Microbiol Rev 1991 55 3 476 511 1943998 10.1128/mr.55.3.476-511.1991 PMC372831

[B20] Wiedmann M Bruce JL Keating C Johnson AE McDonough PL Batt CA Ribotypes and virulence gene polymorphisms suggest three distinct Listeria monocytogenes lineages with differences in pathogenic potential Infect Immun 1997 65 7 2707 2716 9199440 10.1128/iai.65.7.2707-2716.1997 PMC175382

[B21] McNeil LK Reich C Aziz RK Bartels D Cohoon M Disz T Edwards RA Gerdes S Hwang K Kubal M The National Microbial Pathogen Database Resource (NMPDR): a genomics platform based on subsystem annotation Nucleic Acids Res 2007 35 Database D347 353 10.1093/nar/gkl947 17145713 PMC1751540

[B22] Kurtz S Phillippy A Delcher AL Smoot M Shumway M Antonescu C Salzberg SL Versatile and open software for comparing large genomes Genome Biol 2004 5 2 R12 10.1186/gb-2004-5-2-r12 14759262 PMC395750

[B23] Garey MR Johnson DS Computers and Intractability: A Guide to the Theory of NP-Completeness 1979 New York, NY, USA: W. H. Freeman & Co

[B24] Ausiello G Protasi M Marchetti-Spaccamela A Gambosi G Crescenzi P Kann V Complexity and Approximation: Combinatorial Optimization Problems and Their Approximability Properties 1999 Secaucus, NJ, USA: Springer-Verlag New York, Inc

[B25] Johnson D Approximation algorithms for combinatorial problems Proceedings of the fifth annual ACM symposium on Theory of computing 1973 ACM New York, NY, USA 38 49

[B26] Feige U A threshold of ln n for approximating set cover Journal of the ACM (JACM) 1998 45 4 634 652 10.1145/285055.285059

[B27] Toledo-Arana A Dussurget O Nikitas G Sesto N Guet-Revillet H Balestrino D Loh E Gripenland J Tiensuu T Vaitkevicius K The Listeria transcriptional landscape from saprophytism to virulence Nature 2009 459 7249 950 956 10.1038/nature08080 19448609

[B28] The Vmatch large scale sequence analysis software http://www.vmatch.de

[B29] Ergatis http://ergatis.sourceforge.net

[B30] R: A Language and Environment for Statistical Computing http://www.R-project.org

[B31] Tettelin H Riley D Cattuto C Medini D Comparative genomics: the bacterial pan-genome Curr Opin Microbiol 2008 11 5 472 477 10.1016/j.mib.2008.09.006 19086349

[B32] Phillippy AM Mason JA Ayanbule K Sommer DD Taviani E Huq A Colwell RR Knight IT Salzberg SL Comprehensive DNA signature discovery and validation PLoS Comput Biol 2007 3 5 e98 10.1371/journal.pcbi.0030098 17511514 PMC1868776

[B33] Barrett T Troup DB Wilhite SE Ledoux P Rudnev D Evangelista C Kim IF Soboleva A Tomashevsky M Marshall KA NCBI GEO: archive for high-throughput functional genomic data Nucleic Acids Res 2009 37 Database D885 890 10.1093/nar/gkn764 18940857 PMC2686538

[B34] Olshen AB Venkatraman ES Lucito R Wigler M Circular binary segmentation for the analysis of array-based DNA copy number data Biostatistics 2004 5 4 557 572 10.1093/biostatistics/kxh008 15475419

[B35] Willenbrock H Fridlyand J A comparison study: applying segmentation to array CGH data for downstream analyses Bioinformatics 2005 21 22 4084 4091 10.1093/bioinformatics/bti677 16159913

[B36] Pinto FR Aguiar SI Melo-Cristino J Ramirez M Optimal control and analysis of two-color genomotyping experiments using bacterial multistrain arrays BMC Genomics 2008 9 230 10.1186/1471-2164-9-230 18489741 PMC2410139

[B37] Snipen L Nyquist OL Solheim M Aakra A Nes IF Improved analysis of bacterial CGH data beyond the log-ratio paradigm BMC Bioinformatics 2009 10 1 91 10.1186/1471-2105-10-91 19298668 PMC2679023

[B38] Snipen L Repsilber D Nyquist L Ziegler A Aakra A Aastveit A Detection of divergent genes in microbial aCGH experiments BMC Bioinformatics 2006 7 181 10.1186/1471-2105-7-181 16573812 PMC1563484

[B39] Slezak T Kuczmarski T Ott L Torres C Medeiros D Smith J Truitt B Mulakken N Lam M Vitalis E Comparative genomics tools applied to bioterrorism defence Brief Bioinform 2003 4 2 133 149 10.1093/bib/4.2.133 12846395

[B40] Tembe W Zavaljevski N Bode E Chase C Geyer J Wasieloski L Benson G Reifman J Oligonucleotide fingerprint identification for microarray-based pathogen diagnostic assays Bioinformatics 2007 23 1 5 13 10.1093/bioinformatics/btl549 17068088

[B41] Porreca GJ Zhang K Li JB Xie B Austin D Vassallo SL LeProust EM Peck BJ Emig CJ Dahl F Multiplex amplification of large sets of human exons Nat Methods 2007 4 11 931 936 10.1038/nmeth1110 17934468

[B42] Okou DT Steinberg KM Middle C Cutler DJ Albert TJ Zwick ME Microarray-based genomic selection for high-throughput resequencing Nat Methods 2007 4 11 907 909 10.1038/nmeth1109 17934469

[B43] Albert TJ Molla MN Muzny DM Nazareth L Wheeler D Song X Richmond TA Middle CM Rodesch MJ Packard CJ Direct selection of human genomic loci by microarray hybridization Nat Methods 2007 4 11 903 905 10.1038/nmeth1111 17934467

